# Room Temperature Deposition of Crystalline Nanoporous ZnO Nanostructures for Direct Use as Flexible DSSC Photoanode

**DOI:** 10.1186/s11671-016-1437-2

**Published:** 2016-04-26

**Authors:** Byung Suh Han, Salim Caliskan, Woonbae Sohn, Miyoung Kim, Jung-Kun Lee, Ho Won Jang

**Affiliations:** Department of Materials Science Engineering, Research Institute of Advanced Materials, Seoul National University, Seoul, 151-744 South Korea; Department of Mechanical Engineering and Materials Science, University of Pittsburgh, Pittsburgh, PA 15261 USA

**Keywords:** Zinc oxide (ZnO), Room temperature, Dye-sensitized solar cell, Pulsed laser deposition (PLD), Electron recombination

## Abstract

A facile approach to fabricate dye-sensitized solar cells (DSSCs) is demonstrated by depositing (001) oriented zinc oxide (ZnO) nanostructures on both glass and flexible substrates at room temperature using pulsed laser deposition. Unique crystallographic characteristics of ZnO combined with highly non-equilibrium state of pulsed laser-induced ablated species enabled highly crystalline ZnO nanostructures without aid of any chemically induced additives or organic/inorganic impurities at room temperature. Film morphology as well as internal surface area is tailored by varying ambient oxygen pressure and deposition time. It is revealed that the optimization of these two experimental factors was essential for achieving structure providing large surface area as well as efficient charge collection. The DSSCs with optimized ZnO photoanodes showed overall efficiencies of 3.89 and 3.4 % on glass and polyethylene naphthalate substrates, respectively, under AM 1.5G light illumination. The high conversion efficiencies are attributed to elongated electron lifetime and enhanced electrolyte diffusion in the high crystalline ZnO nanostructures, verified by intensity-modulated voltage spectroscopy and electrochemical impedance measurements.

**Electronic supplementary material:**

The online version of this article (doi:10.1186/s11671-016-1437-2) contains supplementary material, which is available to authorized users.

## Background

Flexible, light weight dye-sensitized solar cells (DSSCs) based on plastic substrates are one of the most attractive topics in the field of renewable energy [1]. Incorporating such flexible substrates would allow light weight, shock-resistant power conversion devices [2]. Typical DSSC is consisted of nanoporous film of interconnected TiO_2_ nanoparticles that provide large internal surface area for dye adsorption, interpenetration for liquid redox electrolyte, and pathway for charge transport. However, the fabrication of such nanoparticle-based film requires high temperature annealing of the mesoporous photoanodes films at 400–500 °C in order to improve its crystallinity, interconnection between particles and to eliminate residual organic substances. The annealing step has prevented the utilization of plastic substrates to DSSCs where elevated temperature above 200 °C becomes problematic [3]. Zinc oxide (ZnO)-based photoanodes have been studied in this context as a candidate to replace TiO_2_ nanoparticle films which are dominantly used in DSSCs. While the band gap of ZnO is similar to that of TiO_2_, ZnO exhibits higher electron mobility and lower crystallization temperature than that of TiO_2_. Intrinsically anisotropic crystal structure of ZnO hexagonal wurzite (P6_3_mc) are suitable for low temperature synthesis of the mesoporous films which rapidly collect photogenerated electrons from surrounding dye molecules and minimize the carrier recombination [4–8].

Recently, significant progresses were made in tailoring the morphology and surface properties of nanostructured ZnO. With different surface energies between crystallographic planes, a wide range of high crystalline ZnO nanostructures has been fabricated rather easily at mild conditions compared to other wideband gap metal oxide materials. However, with increased surface-to-volume ratio, ZnO nanostructures are very sensitive to the growth condition and chemical environment, often lacking reproducibility and controllability [9–12]. It has been challenging to prepare efficient ZnO photoanode with enlarged surface area at low temperature because the synthesis of high crystalline nanoparticles and the formation of mesoporous structure with good electronic connectivity between particles were rather challenging. Various alternative methods such as cathodic electrodeposition [13], chemically activated solution process [14], direct metal transfer [15], compression method [16], water vapor treatment [17], and hydrothermal growth [18] have been developed as viable methods at low temperature. Although the highest overall conversion efficiencies among flexible ZnO devices were in the range of 3.1–3.8 %, further treatments were required in order to remove aggregation with structural guiding agents (cathodic electrodeposition), to dissolve surface chemisorbed species (chemical activation), or to achieve crystallinity before being transferred to flexible substrate. To the best of our knowledge, there has been no report on demonstrating one-step room temperature synthesis method of self-assembled nanostructured photoanodes with enlarged surface area so far. By using pulsed laser deposition (PLD), a versatile deposition method using highly energetic-ablated species, fabrication process can be free from organic residues and stoichiometry of the material can be reliably controlled.

In this study, we demonstrate synthesis route of *c*-axis-oriented ZnO nanostructures at room temperature obtaining wurzite structure directly on transparent conducting oxide (TCO) substrate. With the aid of instantaneous, non-equilibrium nature of excimer laser-ablated species combined with anisotropic nature of wurzite phase, high-crystalline naonoporous ZnO films were realized on to glass and flexible substrates alike. Morphology and thickness of nanoporous ZnO films were tailored by controlling deposition parameters of pulsed laser deposition under ambient oxygen environment. Morphology of ZnO photoanodes are found to be strongly correlated to photoelectrochemical properties of the fabricated DSSCs as dye adsorption and electrolyte diffusion in the nanostructures are enhanced. The DSSC with the optimized ZnO photoanode on polyethylene naphthalate substrate show an overall efficiency of 3.4 % under AM 1.5G light illumination which is among the best photon to electron conversion efficiency for devices fabricated at room temperature.

## Methods

ZnO ceramic target for laser ablation was prepared from ZnO powders purchased from Sigma Aldrich (Puriss. p.a. ACS reagent grade) using solid state reaction. Target was pressed in pellet shape and sintered at 800 °C for 2 h. For ZnO photoanodes, tin-doped indium oxide (ITO) glass (Samsung Corning, 8.3 ohm sq^−1^, Korea) and PEN (Peccell, 13 ohm sq^−1^, Japan) were used as the substrates; 0.3 mM N719 dye anhydrous acetonitrile-tert butanol 1:1 solution was prepared for dye sensitization. N719 dye ((2,2ʹ bipyridyl-4,4ʹ dicarboxilate)_2_(NCS)_2_) was obtained from Solaronix. Electrolyte used for dye-sensitized solar cell fabrication is commercially available Merck (SI16L1535-01) iodide-based liquid electrolyte. Anhydrous acetonitrile and tert butanol was obtained from Sigma Aldrich. Pt-sputtered FTO (TEC8, 8 ohm sq^−1^, Pilkington) was used for counter electrode.

Target surface was exposed to concentrated laser pulses by an optic lense. ZnO structure was prepared on both rigid glass and flexible polymer substrates. ZnO target was ablated with KrF excimer laser (248 nm, 1.5 J cm^−2^, 5 Hz, Compex Pro 205F, Coherent, USA). At room temperature, ambient oxygen pressure of high purity O_2_ gas was varied from 100 to 400 mTorr. The thicknesses of nanostructured ZnO films were controlled by deposition time (number of laser pulses) and they were measured by a scanning electron microscope. As-deposited ZnO films were immersed in an N719 solution for 2 h at 50 °C (see Additional file 1: Figure S1, Table S1) [19–21]. Sandwich-type cells were sealed by pressing heat-melted Surlyn tape between sensitized ZnO photoanode and Pt counter electrode. After 3 h, the fabricated cells have shown saturated efficiencies (see Additional file 1: Figure S2, Table S3).

Photovoltaic properties were measured using a potentiostat (CHI 608C, CH instrument, USA) under AM 1.5G, 100 mW light illumination generated by solar simulator (PEC-L11, Peccel, Japan) as well as dark condition. The light intensity of the solar simulator was calibrated with a reference cell (PV Measurements, USA). An electrochemical workstation (Zennium, Zahner, USA) with an attached frequency response analyzer and a light-emitting diode (667 nm) was utilized for the intensity-modulated photovoltage spectroscopy study. A specially designed system (K3100, McScience) was used to obtain incident photon-to-current efficiency (IPCE) in the range 300–800 nm. Electrochemical impedance spectroscopy (EIS) of the cells was also measured using the potentiostat under illumination and applying open-circuit voltage as the bias. Amount of dye adsorbed was determined by desorbing sensitized film in 10 ml 0.1 M NaOH aqueous solution and optical absorption spectra was measured using UV-vis spectrophotometer (Cary 5000, Agilent technologies, USA). X-ray diffraction (XRD; D8-advance, Bruker Miller Co., USA) was used to determine crystalline structure, and film morphology was observed with field emission scanning microscope (FESEM; FSM-6330F, FEOL, Japan). High-resolution transmission electron microscopy (HRTEM) and selective area electron diffraction (SAED) with JEOL JEM-2100F microscope at an acceleration voltage of 200 kV were carried out to investigate the microstructure of the ZnO films.

## Results and Discussion

**Fig. 1:**
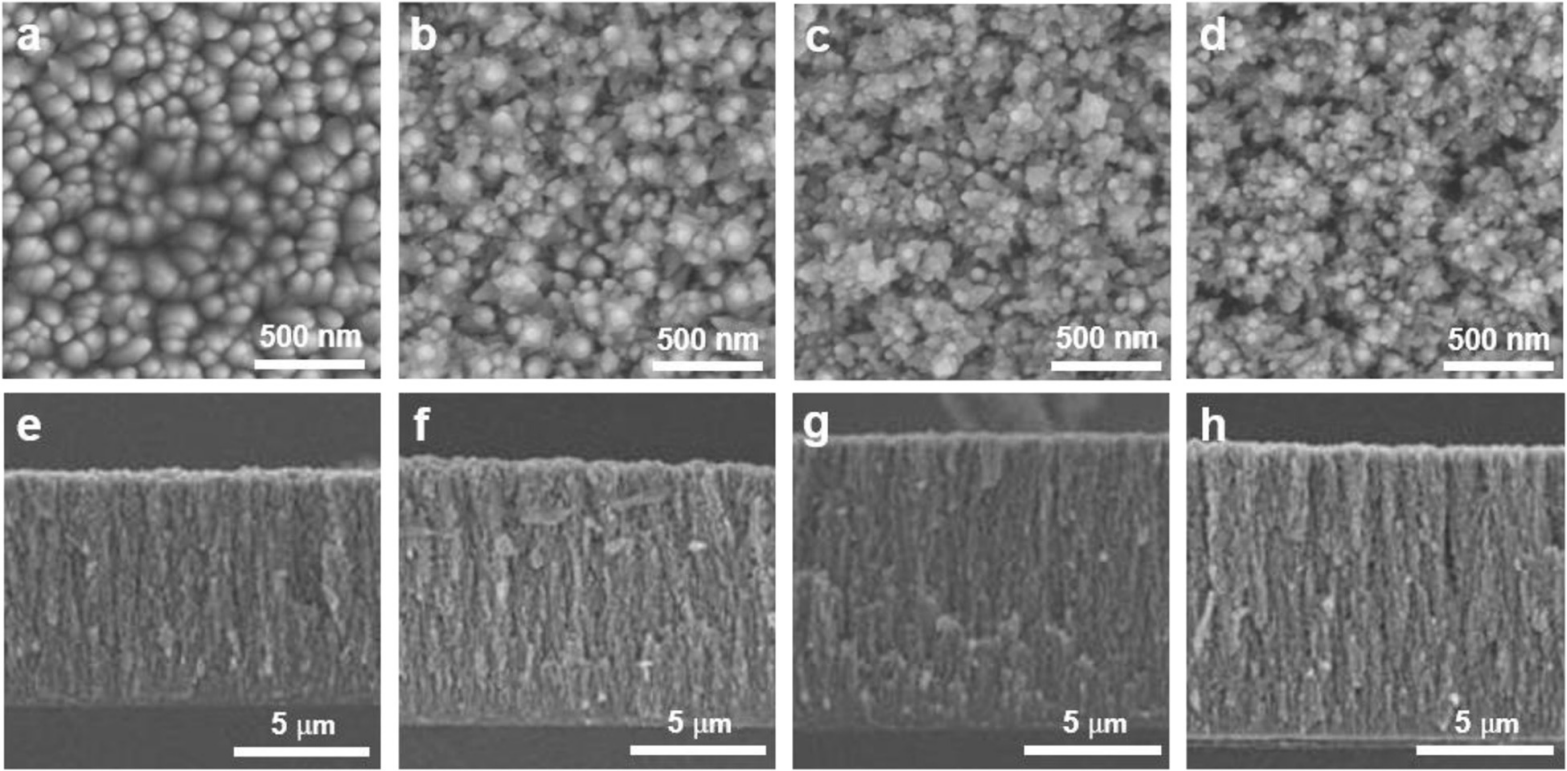
Cross-sectional and plane-view FESEM images of nanostructured ZnO photoanodes deposited under various ambient oxygen pressures on ITO/glass substrates. **a**, **e** 100 mTorr. **b**, **f** 200 mTorr, **c**, **g** 300 mTorr. **d**, **h** 400 mTorr

Morphology transition of PLD-generated ZnO film on ITO glass substrate tuned with the function of ambient gas pressure can be seen in Fig. 1. Ablated species lose the kinetic energy as it propagates through the ambient oxygen gas in the PLD chamber, colliding with the oxygen molecules. The remaining kinetic energy reaching the substrate surface determines the film porosity and alignment. By controlling the deposition parameters such as oxygen pressure and target to substrate distance, the morphology of photoanodes could be systematically controlled [22].

Deposited films show hierarchical morphology constructed with 100-nm-sized ZnO nanoparticles, while porosity of the film changes as gas pressure inside the chamber is tuned. Morphology transition from dense, cone-like structure at 100 mTorr to porous open structure at higher ambient gas pressure (200–400 mTorr) enabled large internal surface area where the local maximum was obtained at 300 mTorr. All as-prepared samples showed P63mc wurzite phase of ZnO without further treatment.

**Fig. 2:**
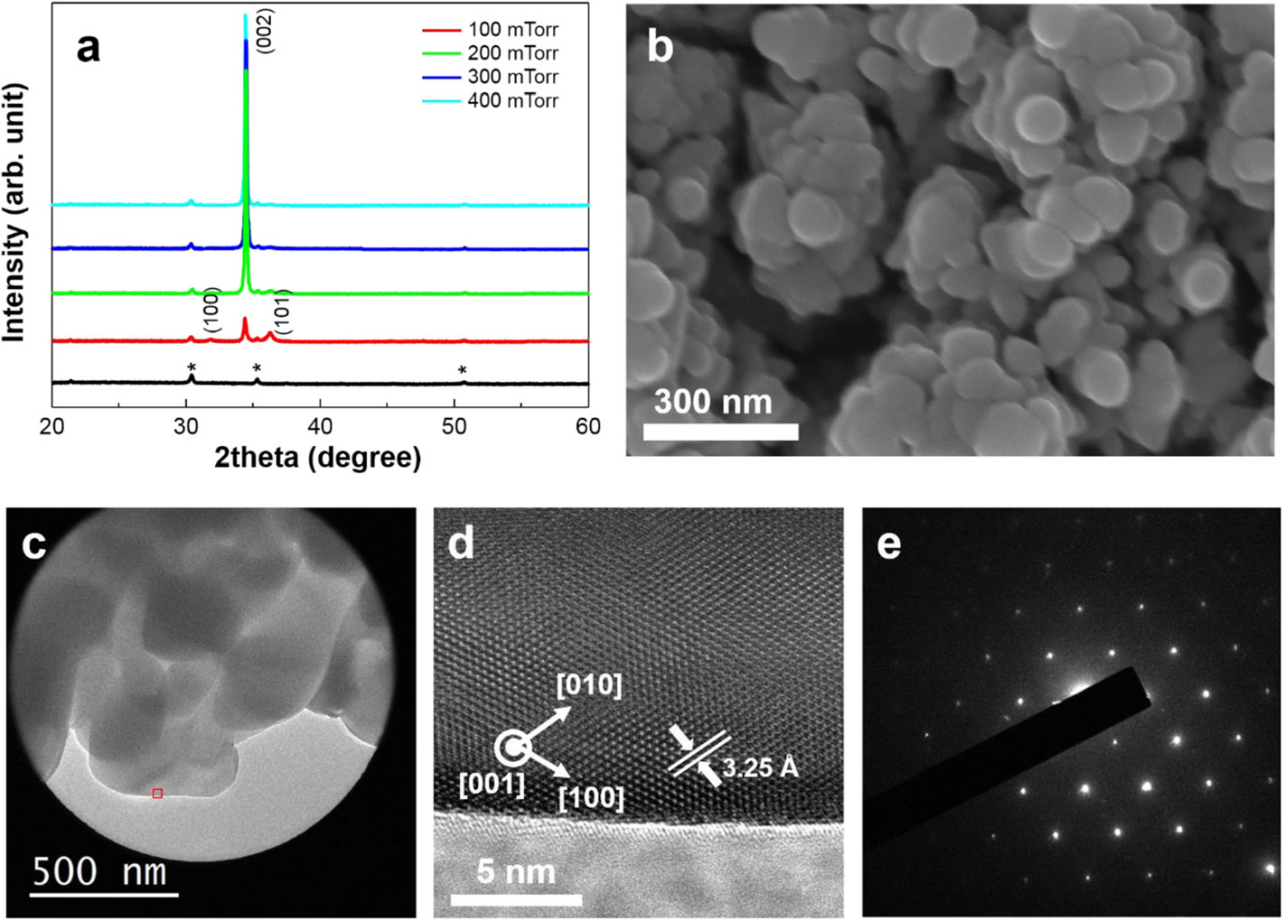
**a** XRD patterns of nanostructured ZnO photoanodes deposited on ITO/glass substrates as a function of the ambient oxygen pressure. The *black line* represents diffraction pattern of ITO substrate. Diffraction peaks of ITO are marked as *asterisk*. **b** High-magnification plane view SEM image of the ZnO photoanode deposited under 300 mTorr. **c** TEM image of the ZnO photoanode deposited under 300 mTorr. **d** High-resolution TEM image of the ZnO nanoparticle from the marked area in **c**. **e** Selective area electron diffraction pattern of the ZnO photoanode

It is notable that PLD generated nanostructures are constructed with necked spherical nanoparticles, which seem randomly attached, show strong (001) oriented crystal structure according to our XRD results (Fig. 2a). FESEM and TEM were employed to further examine the microstructure and morphology of the ZnO photoanodes. Figure 2b, c clearly shows that spherical ZnO nanoparticles with diameters around 100 nm are the building blocks of the porous structures. The high-magnification SEM image clearly shows that the ZnO film is nanoporous with open channels that have varied width from tens of nanometers to hundreds of nanometers. We believe that the vertical channels act as diffusion paths for the electrolyte. The TEM image taken from the nanostructure indicates that each particle is a well-crystallized hexagonal wurzite phase (Fig. 2d), and the SEAD pattern confirmed that crystalline ZnO particles are preferentially oriented along the *c*-axis direction (Fig. 2e). We believe that PLD plume generated by instantaneous energy concentration of nanosecond excimer laser pulse created strong driving force for minimizing high energy polar plane of (001) where uncompensated surface net charge is not zero during MS-scaled time of flight until they reach substrate surface followed with deposition process [23]. The results we obtained from PLD-derived nanostructures can be compared with randomly oriented nanostructures (nanoparticle based) which were synthesized by conventional screen printing method accompanied with post annealing process using pre-synthesized nanoparticle paste which contained organic binder. Reported experimental results as well as our own suggest that the XRD pattern of randomly oriented nanoparticle-based structure is similar to that of randomly oriented polycrystalline ZnO particle, indicating that necking direction between pre-synthesized nanoparticles during annealing process is random and follows their geometrical state as they were screen printed.

Nanocluster generation during PLD has been investigated until recently [24]. However, exact mechanism under ambient gas pressure has yet to be fully understood. By combining insights earned from our experimental results and reviewing theoretical and fundamental studies so far, we suggest the formation mechanism of oriented nanostructure deposition during PLD. Ablated species ejected from the target surface induced by concentrated excimer laser pulses initially forms highly concentrated, a non-equilibrium gas state called Knudsen layer, followed by rapid vapor plume expansion pushing ambient gas away. Expanded gas is then pushed back and compressed. Nanoparticles are reported to be concentrated in the region between plume and buffer gas where the highest supersaturation is reached [25, 26] due to the extremely fast quenching rate right after the initial ablation. High spatial localization of nanoparticles leads to three-dimensional fractal aggregations kinetically deposited onto the substrate forming columnar nanostructures suggesting diffusion limited aggregation [27] is the main factor when cluster formation takes place during PLD plume condensation and deposition.

**Fig. 3:**
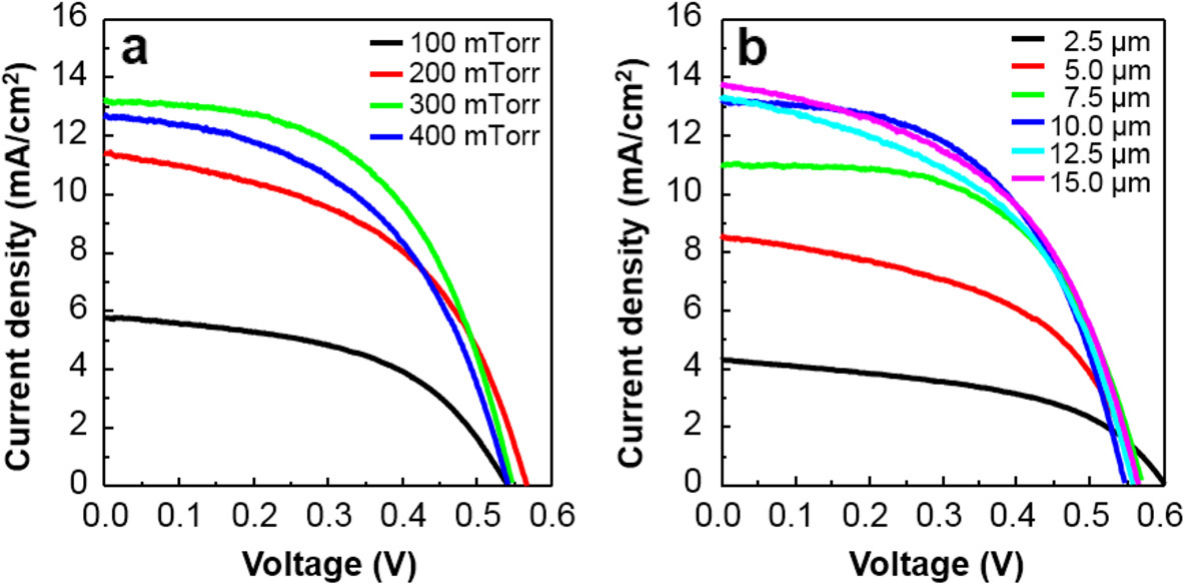
**a** *J–V* curves of DSSCs fabricated with nanostructured ZnO photoanodes deposited under different ambient oxygen pressures. The thickness of the photoanodes was fixed to be 10 μm. **b** *J–V* curves of DSSCs fabricated with nanostructured ZnO photoanodes of different thicknesses. All photoanodes were deposited under oxygen pressure of 300 mTorr

**Fig. 4:**
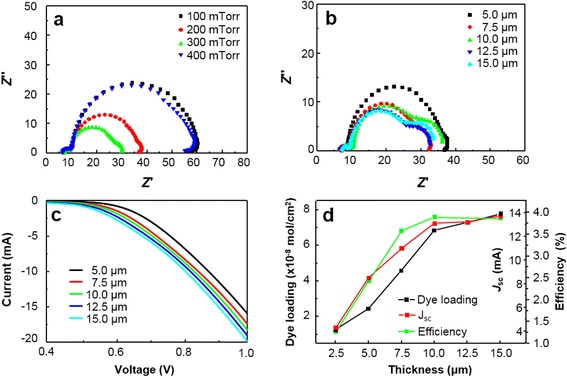
**a** Nyquist plots of DSSCs with nanostructured ZnO photoanodes deposited under different ambient oxygen pressures. The thickness of the photoanodes was fixed to be 10 μm. **b** Nyquist plots of DSSCs with nanostructured ZnO photoanodes of different thicknesses. All photoanodes were deposited under oxygen pressure of 300 mTorr. **c** Dark current characteristics of the DSSCs with nanostructured ZnO photoanodes of different thicknesses. **d** Short-circuit current density (*J*_sc_), the amount of dye loading, and the net efficiency for the DSSCs with nanostructured ZnO photoanodes of different thicknesses

**Fig. 5:**
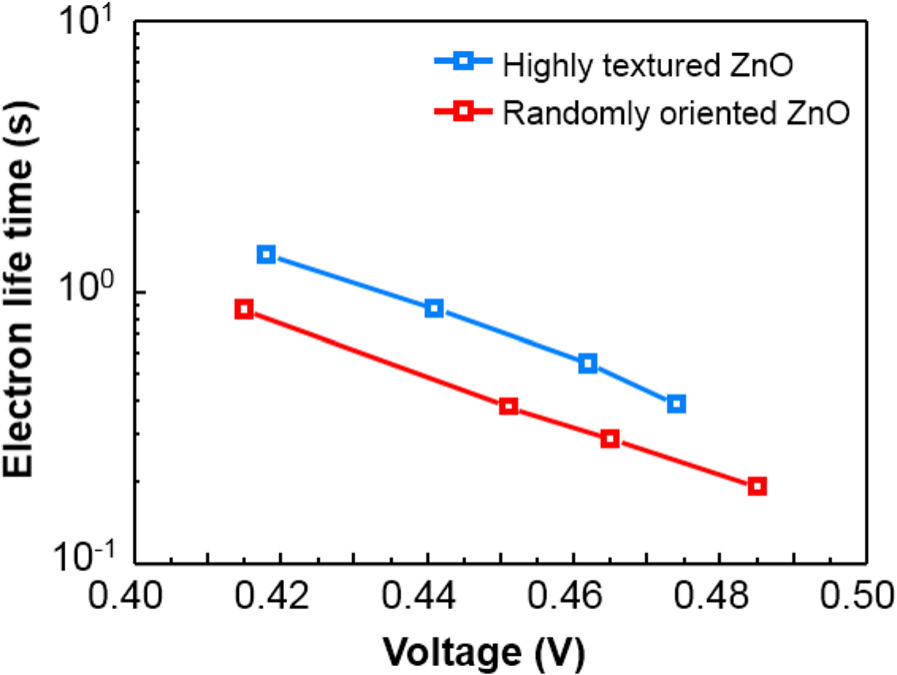
Electron lifetime measured by IMVS as a function of the open circuit voltage for DSSCs fabricated with pulsed laser deposited (highly textured) and nanoparticle-based (randomly oriented) ZnO photoanodes

Photocurrent density-photovoltage (*J*–*V*) curves of PLD-derived ZnO DSSCs varying ambient gas pressure and film thickness are shown in Fig. 3 and Table 1. PLD-derived ZnO samples were deposited at the pressure range of 100–400 mTorr and no further treatment was applied to deposited layers and the cells were measured under AM 1.5G simulated light illumination. The conversion efficiency of the devices is strongly correlated by the deposition pressure and the film thickness. *J*_sc_ shows strong correlation with ambient gas pressure during deposition, changing from 5.6 to 13.1 mA cm^−2^. The film deposited at 300 mTorr exhibits the highest *J*_sc_ (13.1 mA cm^−2^) and efficiency (3.81 %) at the 10-μm film thickness. Using the optimized condition, we fabricated five different cells. They showed quite uniform *J*_sc_ and efficiency values (see Additional file 1: Table S3, Figure S3). This result suggests that the overall conversion efficiency (*η*) is strongly correlated with *J*_sc_, which is also strongly corrrelated with the amount of dye loading on the surface of the photoanode. The maximum IPCE was measured to be about 58 % at 530 nm (see Additional file 1: Figure S4). The effect of morphology tuning on the surface area and net conversion efficiency is investigated with EIS. *J*_sc_ is highly correlated by the amount of dye loading which is proportional to the specific internal surface area of the deposited nanostructures (see Additional file 1: Table S2). The actual surface area occupied by a dye molecule can be obtained based on the assumption that single N719 dye occupies area around 1 nm^2^ as its N3 dye counterpart [28, 29]. EIS data for DSSCs with the ZnO photoanodes grown under different ambient oxygen pressures (at the fixed thickness) and of different thicknesses (under the same ambient oxygen pressure) are shown in Fig. 4a, b, respectively. The high frequency region higher than 146.5 Hz is related to the sheet resistance of the TCO layer. The frequency regions of 97.7 to 117.2 Hz (*ω*_1_), 1.18 to 97.7 Hz (*ω*_2_), and 0.118 to 1.18 Hz (*ω*_3_) are respectively associated with the charge transport at the ZnO/TCO layer or Pt/electrolyte interfaces, the ZnO/dye/electrolyte interface, and the Nernstian diffusion of the electrolyte. From EIS data in Fig. 4a, it is suggested that the largest surface area is achieved at 300 mTorr where the radius of *Z*_2_ is minimized. As ambient gas pressure is tuned from 100 to 400 mTorr, the resultant ZnO film changes from a dense closed structure to a porous open structure. As the surface area of the film is increased, dye adsorption and electrolyte diffusion can be also increased. From Fig. 4b, it is revealed that surface area is proportional to the film thickness as the radius of *Z*_2_ increases with the film thickness. Figure 4c shows the dark *J*–*V* curves of the fabricated cells with different ZnO thicknesses. It reveals that dark current (i.e., back electron transfer) increases as the thickness and the surface area increase. This result indicates that the use of the photoanode with the optimized thickness is needed to suppress the recombination of photogenerated electrons with the electrolyte, which is consistent with the photovoltaic measurements and the EIS analysis. However, overall efficiency saturation at the longer structures (12.5 and 15 μm) indicates trade-off relations between the amount of dye loading and back electron transfer (recombination) reaction and between the amount of dye loading and electrolyte diffusion. For further investigation of our PLD-generated, highly textured ZnO nanostructures, electron recombination time constant (IMVS, intensity-modulated voltage spectroscopy) as a function of the open circuit voltage is compared with conventional screen printed randomly oriented sample sintered at 450 °C. Results are shown in Fig. 5 that highly textured ZnO nanostructures has an electron lifetime which is one order of magnitude longer than that of a random oriented paste-based ZnO nanostructures. The enhanced electron lifetime of the highly textured DSSCs can be ascribed to the vertically oriented porous structure and (001) oriented crystallinity of the photoanodes. I_3_^−^ ions are generated at dye/electrolyte interface when I^−^ ions are oxidized during the dye regeneration process. I^−^ ions need to diffuse through the nanostructures to the Pt cathode surface, and the average diffusion length is approximated to be the half of the film thickness. However, vertically aligned ZnO nanostructured photoanodes provide fast diffusion channels through the pore network resulting in lower equilibrium I_3_^−^ ion concentration compared to the nanoparticle-based photoanode, enhancing the lifetime of injected electrons inside the oxide network [30, 31].

**Table 1:** Photovoltaic parameters of DSSCs with nanostructured ZnO photoanodes under simulated AM 1.5 G light illumination

A				
	*J*_sc_ (mA cm^−2^)	*V*_oc_ (V)	FF (%)	*η* (%)
100 mTorr	508	0.44	51	1.58
200 mTorr	11.4	0.57	50	3.23
**300 mTorr**	**13.1**	**0.55**	**54**	**3.89**
400 mTorr	12.7	0.53	51	3.41
B				
2.5 μm	4.3	0.60	49	4.27
5.0 μm	8.5	0.57	50	2.43
7.5 μm	11	0.57	57	3.58
**10.0 μm**	**13.1**	**0.55**	**54**	**3.89**
15.0 μm	13.7	0.56	50	3.86
Flexible 10 μm	10.8	0.55	57	3.4

*J*_sc_, *V*_oc_, FF, and *η* denote saturated photocurrent, open circuit voltage, fill factor, and overall efficiency, respectively

*A* as a function of ambient oxygen pressure for ZnO deposition (the thicknesses of all the ZnO films were fixed to be 10 μm)

*B* as function of film thickness (all films were deposited under 300 mTorr)

**Fig. 6:**
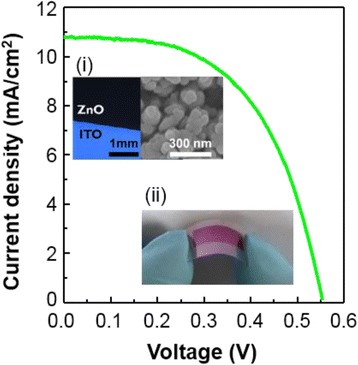
*J–V* relation of the DSSC with a nanostructured ZnO photoanode fabricated on a flexible ITO/PEN substrate. The *insets* show the (*i*) optical microscope image and high-magnification plane view FESEM image of a 10-μm-thick ZnO film grown at 300 mTorr on flexible ITO/PEN substrate and (*ii*) a photograph of the flexible DSSC

Figure 6 shows *J*–*V* characteristic of flexible photovoltaic fabricated on ITO/PEN substrate. Sample was prepared at optimized condition for DSSCs in ITO/glass system (300 mTorr, 10 μm). The conversion efficiency of the device is lower compared to ITO/glass-based device because of the higher resistivity and the lower optical transparency of the ITO/PEN substrate (see Additional file 1: Figure S5). The versatile characteristic of PLD-derived ZnO photonodes is demonstrated. It is implied that identical nanostructures of similar device performance can be fabricated using our PLD method. We believe that further usage of the deposition method will find significant applications in photovoltaic and other electrochemical applications and open up an opportunity in understanding the mechanism of self-assembled nanostructure formation during PLD.

## Conclusions

In conclusion, we demonstrate the direct synthesis of ZnO nanostructures on ITO/glass and ITO/polymer substrates with controllable surface area using PLD method. All films showed crystalline ZnO wurzite phase as deposited at room temperature and morphology of the nanostructured film were tuned with the function of ambient gas pressure. *J*_sc_ which is strongly correlated with surface area for dye adsorption sites was tuned out to be the most important parameter determining overall conversion efficiency. The optimal conversion efficiency of 3.89 % was achieved under AM 1.5 G light illumination on ITO/glass substrate and 3.4 % on ITO/PEN flexible substrate, which is the highest among flexible, room temperature-fabricated ZnO DSSCs. Our results suggest the versatility of PLD combined with anisotropic characteristic of ZnO resulted in successful synthesis of crystalline nanostructures providing well-necked, oriented nanostructures with enlarged surface area at room temperature, showing promising potential uses for other electrochemical applications and materials as well.

## Additional Files

Additional file 1: Figure S1.
*J*–*V* curves for four different ZnO electrodes with different dye and solution combination in 2 h sensitizing time. **Figure S2.** (a) *J–V* curves of DSSCs fabricated with nanostructured ZnO photoanodes as a function of dye adsorption time at 50 °C (all films were deposited under 300 mTorr and the thicknesses of all films were fixed to be 6.7 μm) and (b) as function of sample aging after fabrication. **Table S1.** Device parameters of dye-sensitized ZnO nanostructured photoanodes under simulated AM 1.5 G light illumination (a) as a function of dye adsorption time at 50 °C (the thicknesses of the films were fixed to be 6.7 μm) and (b) as function of sample aging after fabrication. **Table S2.** Dye loading of DSSCs fabricated with nanostructured ZnO photoanodes deposited under different ambient oxygen pressures. The thickness of the photoanodes was fixed to be 10 μm. **Table S3.** Statistical analysis of device parameters for five different DSSCs fabricated with nanostructured ZnO photoanodes deposited by PLD using the optimized condition. **Figure S3.** *J–V* curves of five different DSSCs fabricated with nanostructured ZnO photoanodes deposited by PLD using the optimized condition. **Figure S4.** The incident photon-to-current conversion efficiency (IPCE) spectrum of a DSSC with a nanostructured ZnO photoanode deposited under 300 mTorr by PLD. **Figure S5.** *J–V* curves of 300 mTorr 5-μm ZnO photoanodes deposited by PLD using PLD coupled with Pt/ITO/PEN flexible substrate. (DOCX 226 kb)
