# Supplementary material for: Room Temperature Deposition of Crystalline Nanoporous ZnO Nanostructures for Direct Use as Flexible DSSC Photoanode
Source: Nanoscale Res Lett. 2016 Apr 26;11:221. doi: 10.1186/s11671-016-1437-2 (PMC4844570; doi:10.1186/s11671-016-1437-2)
Supplement: Additional file 1: Figure S1. — J–V curves for four different ZnO electrodes with different dye and solution combination in 2 h sensitizing time. Figure S2. (a) J–V curves of DSSCs fabricated with nanostructured ZnO photoanodes as a function of dye adsorption time at 50 °C (all films were deposited under 300 mTorr and the thicknesses of all films were fixed to be 6.7 μm) and (b) as function of sample aging after fabrication. Table S1. Device parameters of dye-sensitized ZnO nanostructured photoanodes under simulated AM 1.5 G light illumination (a) as a function of dye adsorption time at 50 °C (the thicknesses of the films were fixed to be 6.7 μm) and (b) as function of sample aging after fabrication. Table S2. Dye loading of DSSCs fabricated with nanostructured ZnO photoanodes deposited under different ambient oxygen pressures. The thickness of the photoanodes was fixed to be 10 μm. Table S3. Statistical analysis of device parameters for five different DSSCs fabricated with nanostructured ZnO photoanodes deposited by PLD using the optimized condition. Figure S3. J–V curves of five different DSSCs fabricated with nanostructured ZnO photoanodes deposited by PLD using the optimized condition. Figure S4. The incident photon-to-current conversion efficiency (IPCE) spectrum of a DSSC with a nanostructured ZnO photoanode deposited under 300 mTorr by PLD. Figure S5. J–V curves of 300 mTorr 5-μm ZnO photoanodes deposited by PLD using PLD coupled with Pt/ITO/PEN flexible substrate. (DOCX 226 kb) [file 11671_2016_1437_MOESM1_ESM.docx]

**Additional file**

**Room temperature deposition of crystalline nanoporous ZnO nanostructures for direct use as flexible DSSC photoanode**

Byung Suh Han^a^, Salim Caliskan^b^, Woonbae Sohn^a^, Miyoung Kim^a^, Jung-Kun Lee^b^, Ho Won Jang^a^

*^a^Department of Materials Science Engineering, Research Institute of Advanced Materials, Seoul National University, Seoul 151-744, Korea*

*^b^Department of Mechanical Engineering and Materials Science, University of Pittsburgh, 522 Benedum Hall, Pittsburgh, PA 15261, USA*

Corresponding Authors

*Email: [hwjang@snu.ac.kr](mailto:hwjang@snu.ac.kr); Fax: +82-2-884-1416; Tel: +82-2-880-1720


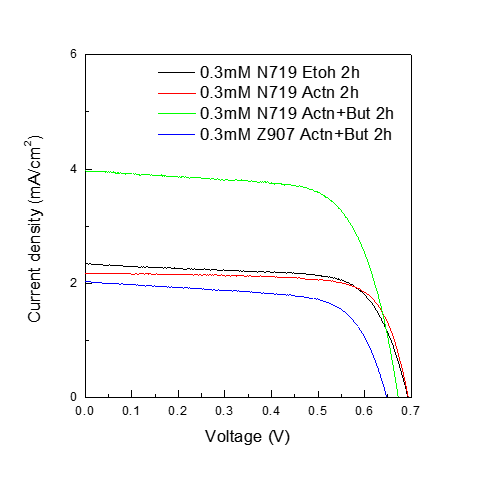


Additional file 1: Figure S1

*J-V* curves for 4 different ZnO electrodes with different dye and solution combination in 2 hour sensitizing time.

Additional file 1: Figure S2

(a) *J-V* curves of DSSCs fabricated with nanostructured ZnO photoanodes as a function of dye adsorption time at 50℃ (all films were deposited under 300 mTorr and the thicknesses of all films were fixed to be 6.7 μm) and (b) as function of sample aging after fabrication.

Additional file 1: Table S1

Device parameters of dye-sensitized ZnO nanostructured photoanodes under simulated AM 1.5G light illumination (a) as a function of dye adsorption time at 50℃ (the thicknesses of the films were fixed to be 6.7 μm) and (b) as function of sample aging after fabrication.


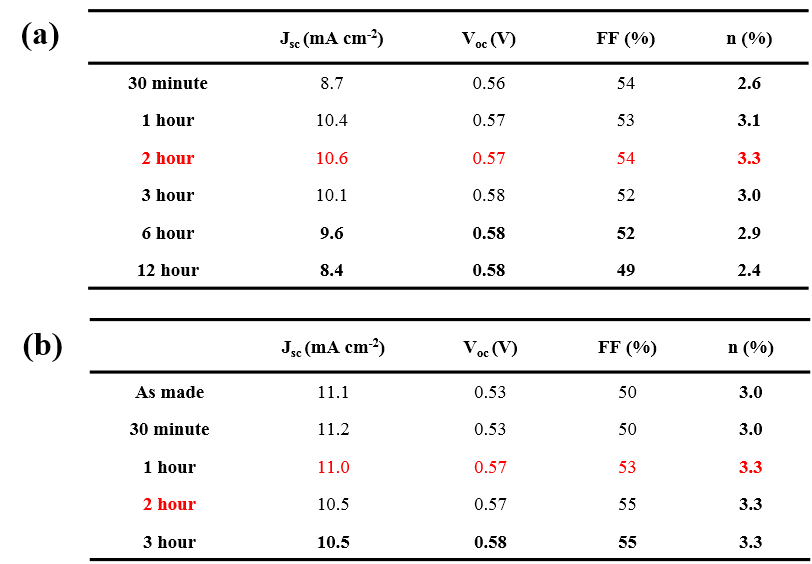


Additional file 1: Table S2

Dye loading of DSSCs fabricated with nanostructured ZnO photoanodes deposited under different ambient oxygen pressures. The thickness of the photoanodes was fixed to be 10 µm.


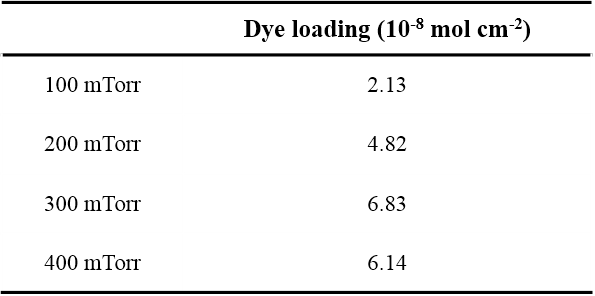


Additional file 1: Table S3

Statistical analysis of device parameters for 5 different DSSCs fabricated with nanostructured ZnO photoanodes deposited by PLD using the optimized condition.

|  | | ***J*_sc_ (mA cm^-2^)** | ***V*_oc_ (V)** | **FF (%)** | ***η* (%)** |
| --- | --- | --- | --- | --- | --- |
| **5 devices on ITO/glass substrates** | **Ave.** | **13.04** | **0.552** | **50** | **3.893** |
|  | **Std. Dev.** | **0.3435** | **0.0130** | **50** | **0.0095** |
|  | **Range** | **12.7−13.1** | **0.54−0.57** | **0.53−0.55** | **3.888−3.910** |

Additional file 1: Figure S3

*J-V* curves of 5 different DSSCs fabricated with nanostructured ZnO photoanodes deposited by PLD using the optimized condition.


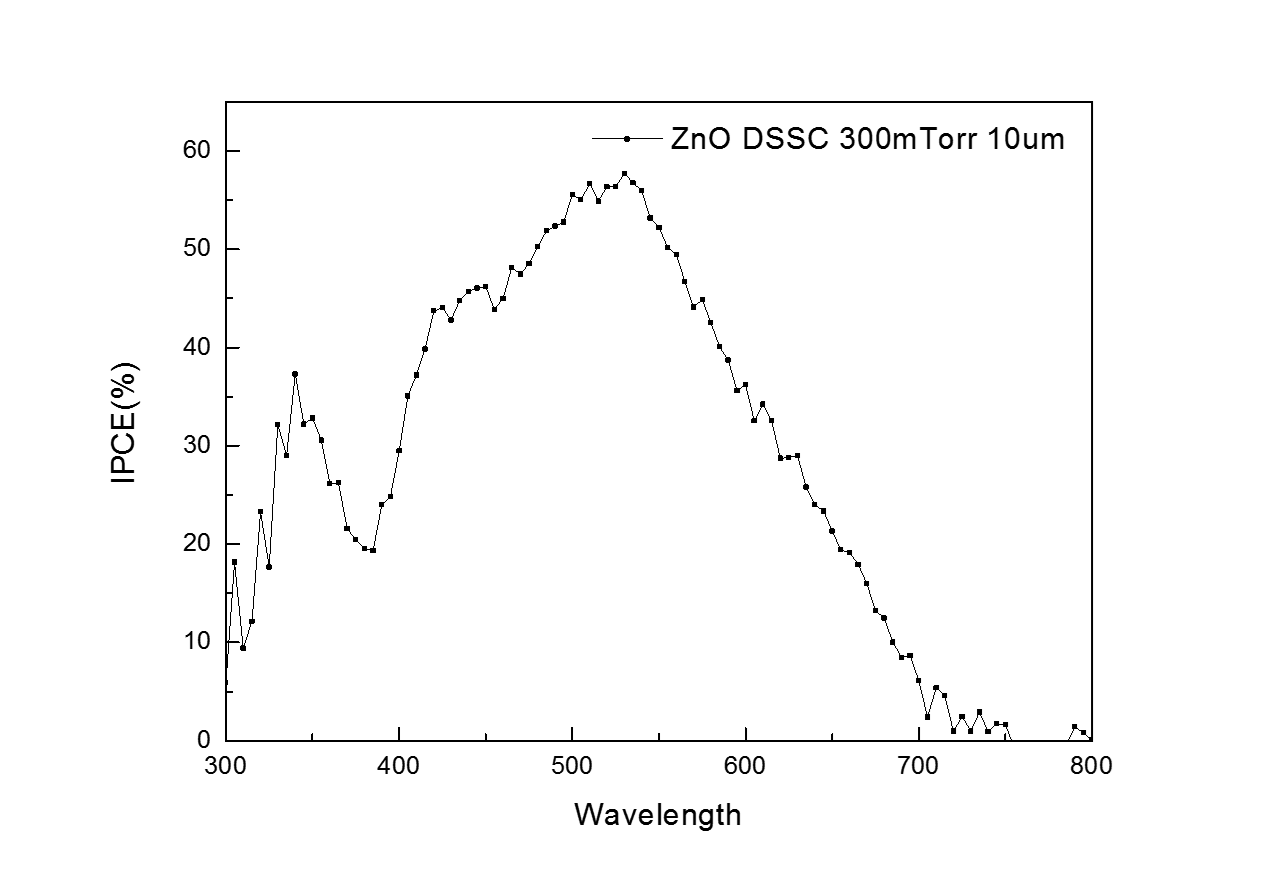


Additional file 1: Figure S4

The incident photon-to-current conversion efficiency (IPCE) spectrum of a DSSC with a nanostructured ZnO photodanode deposited under 300 mTorr by PLD.

**
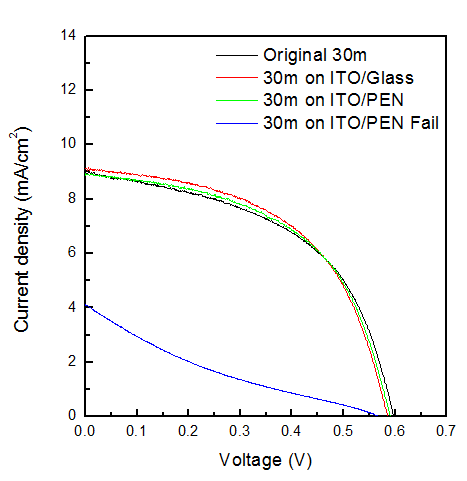
**

Additional file 1: Figure S5

*J-V* curves of 300mTorr 5μm ZnO photoanodes deposited by PLD using PLD coupled with Pt/ITO/PEN flexible substrate.
